# Association between the *XKR6* rs7819412 SNP and serum lipid levels and the risk of coronary artery disease and ischemic stroke

**DOI:** 10.1186/s12872-019-1179-z

**Published:** 2019-08-20

**Authors:** Peng-Fei Zheng, Rui-Xing Yin, Guo-Xiong Deng, Yao-Zong Guan, Bi-Liu Wei, Chun-Xiao Liu

**Affiliations:** 1 Department of Cardiology, Institute of Cardiovascular Diseases, the First Affiliated Hospital, Guangxi Medical University, Nanning, 530021 Guangxi People’s Republic of China; 2 Guangxi Key Laboratory Base of Precision Medicine in Cardio-cerebrovascular Disease Control and Prevention, Nanning, 530021 Guangxi People’s Republic of China; 3 Guangxi Clinical Research Center for Cardio-cerebrovascular Diseases, Nanning, 530021 Guangxi People’s Republic of China

**Keywords:** Coronary artery disease, Ischemic stroke, *XKR6*, Single nucleotide polymorphism, rs7819412, Lipids

## Abstract

**Background:**

The present study aimed to expound the association between the XK related 6 gene (*XKR6*) rs7819412 single nucleotide polymorphism (SNP) and serum lipid profiles and the risk of coronary artery disease (CAD) and ischemic stroke.

**Methods:**

The genetic makeup of the *XKR6* rs7819412 SNP in 1783 unrelated participants (controls, 643; CAD, 588 and ischemic stroke, 552) of Han Chinese was obtained by the Snapshot technology.

**Results:**

The genotypic frequencies of the SNP were disparate between CAD (GG, 81.0%; GA/AA, 19.0%) or ischemic stroke (GG, 81.2%; GA/AA, 18.8%) patients and healthy controls (GG, 85.7%, GA/AA, 14.3%; *P* < 0.05 vs. CAD or ischemic stroke; respectively). The A allele frequency was also diverse between CAD (10.1%) or ischemic stroke (10.0%) and control groups (7.5%; *P* < 0.05 vs. CAD or ischemic stroke; respectively). The GA/AA genotypes and A allele were associated with high risk of CAD and ischemic stroke (CAD: *P* = 0.026 for GA/AA vs. GG, *P* = 0.024 for A vs. G; Ischemic stroke: *P* = 0.029 for GA/AA vs. GG, *P* = 0.036 for A vs. G). The GA/AA genotypes were also associated with increased serum triglyceride (TG) concentration in CAD and total cholesterol (TC) concentration in ischemic stroke patients.

**Conclusions:**

These data revealed that the *XKR6* rs7819412 A allele was related to increased serum TG levels in CAD, TC levels in ischemic stroke patients and high risk of CAD and ischemic stroke.

## Background

Dyslipidemia is a heritable risk factor for coronary artery disease (CAD) which contributed to a prominent reason of disability, mortality, morbidity, functional deterioration and expensive healthcare, and accounts for approximately 30% of all the deaths worldwide [1–3]. Previous studies have shown that CAD occurs due to various factors and can be subjective to genomic background, lifestyle, environmental factors and alterations of plasma lipid levels as well as their interactions with each other [4, 5]. Atherosclerosis is generally considered to be the pathological foundation of CAD [6] and ischemic stroke [7], which is caused by the accumulation of cholesterol in arterial wall macrophages and the dysregulation of metabolic rate of lipids for example increased levels of low-density lipoprotein cholesterol (LDL-C) [8], and apolipoprotein (Apo) B [9], triglyceride (TG) [10], total cholesterol (TC) [11], along with reduced levels of high-density lipoprotein cholesterol (HDL-C) [12] and ApoA1 [9] in serum. In addition, hereditary elements are deemed to account for about 50–80% of the incidence of dyslipidemia [13] and 30–60% of the occurrence of CAD and ischemic stroke [14].

Many genetic loci that are closely associated with lipid metabolism were identified by genome-wide association studies (GWASes), and specific loci among them were also found to be associated with CAD, type 2 diabetes (T2DM), hypertension, and body mass index (BMI) [15]. Previous GWAS has demonstrated that the rs7819412 single nucleotide polymorphism (SNP) near the XKR6 gene (*XKR6*; also knows as: *XRG6; C8orf5; C8orf7; C8orf21*, GeneID: 286046, HGNC: 27806, locus type: gene with protein product, located in chromosome 8p23.1) was associated with TG levels and T2DM in Europeans [16]. Nevertheless, the association between the *XKR6* rs7819412 SNP and blood lipid levels and the risk of CAD and ischemic stroke is not clear and not reported in the Han Chinese. Thus, this study was designed to understand the relationship between the *XKR6* rs7819412 SNP and several serum lipid parameters and the risk of CAD and ischemic stroke in the Han Chinese.

## Methods

### Subjects

A total of 552 unrelated patients with ischemic stroke and 588 participants with CAD were selected from the First Affiliated Hospital of Guangxi Medical University. CAD was defined as significantly coronary artery stenosis (≥ 50%) in at least anyone of the three main coronary vessels or their main branches (branch diameter ≥ 2 mm) [17]. All of the patients with ischemic stroke have received a brain magnetic resonance imaging (MRI) scan and strict neurological examination. The diagnostic criteria for ischemic stroke were derived from the International Classification of Diseases (9th Revision). All subjects with a history of type 1 diabetes, neoplastic, autoimmune, liver, hematologic, thyroid, and renal were excluded. Patients with CAD had no history of ischemic stroke and patients with ischemic stroke also had no history of CAD.

A total of 643 healthy controls matched by ethnic group (Han Chinese), age, gender were also recruited. All subjects were healthy and none of them had a history of CAD, myocardial infarction (MI), ischemic stroke and T2DM as judged by history taking, questionnaires, and critical clinical examination. All participants were randomly collected from the Physical Examination Center of the First Affiliated Hospital, Guangxi Medical University in the same period. Before the beginning of the research, all participants had signed a written informed consent. The research proposal was approved by the Ethics Committee of the First Affiliated Hospital, Guangxi Medical University (No: Lunshen-2011-KY-Guoji-001; Mar. 7, 2011).

### Genotyping and biochemical assays

A venous blood sample of 5 ml was collected from each participant after at least 12 h of fasting. Part of the sample (2 ml) was placed in a glass tube and used to determine serum lipid levels. The remaining sample (3 ml) was collected in the tubes containing anticoagulants (4.80 g/L citric acid, 14.70 g/L glucose, 13.20 g/L tri-sodium citrate) and was utilized to extract deoxyribonucleic acid (DNA). Genotyping of the *XKR6* rs7819412 SNP (rs7819412F: 5′-CGAGTGGTTCTTCCCAGCATGT-3′ and rs7819412R: 5′-ATGTGCCCCCACACCATCATT-3′) was performed by the Snapshot technology [18]. Methods for the detection of serum HDL-C, LDL-C, ApoA1, TC, ApoB, TG levels were referred to our previous study [19]. Serum lipid levels were tested using an autoanalyzer (Type 7170A; Hitachi Ltd., Tokyo, Japan) in the Clinical Science Experiment Center of the First Affiliated Hospital, Guangxi Medical University [20, 21].

### Diagnostic criteria

The levels of serum TG (0.56–1.70 mmol/L), ApoB (0.80–1.05 g/L), TC (3.10–5.17 mmol/L), LDL-C (2.70–3.10 mmol/L), ApoA1 (1.20–1.60 g/L), HDL-C (1.16–1.42 mmol/L), and the ApoA1/ApoB ratio (1.00–2.50) were defined as normal values at our Clinical Science Experiment Center. The diagnostic criteria of hyperlipidemia, hypertension, obesity, normal weight, and overweight were referred to previous studies [19–24]. Somebody who has been previously diagnosed with diabetes or people with 2 h postprandial plasma glucose ≥11.1 mmol/L or a fasting plasma glucose ≥7.0 mmol/L were defined as diabetic patients [25].

### Statistical analyses

All data were evaluated using SPSS (Version 22.0). Values were presented as mean ± SD. Hardy-Weinberg equilibrium was verified by standard goodness of fit test. The chi-square test was used to calculate the genotype distribution between cases and controls. Independent-samples *t* test was used to analyze the difference in general characteristics between patients and controls. The relationship between serum lipid levels and genotypes was tested by covariance analysis (ANCOVA). Gender, age, blood pressure, cigarette smoking, BMI and alcohol consumption were adjusted for the statistical analysis. Unconditional logistic regression analysis was used to detect the odds ratio (OR) and 95% confidence interval (CI). The heart-map of the inter-locus models was measured by R software (version 3.5.3). A *P-*value < 0.05 was considered as statistically significant.

## Results

### Common and biochemical characteristics

As mentioned in Table 1, the ratio of female to male, age, serum ApoB levels and the proportion of smokers were similar between the controls and cases. The height, weight, BMI, systolic blood pressure, glucose, pulse pressure, serum LDL-C, TG and TC levels were significantly lower; and serum HDL-C and ApoA1 levels, the ApoA1/ApoB ratio, and the proportion of drinkers were significantly higher in controls than in CAD and ischemic stroke patients. The level of diastolic blood pressure was lower in ischemic stroke patients as compared with controls.

**Table 1:** Comparison of demographic, lifestyle characteristics and serum lipid levels of the participants

Characteristic	Control (*n* = 643)	Case		*P* vs. *controls*	
		CAD (*n* = 588)	Ischemic stroke (*n* = 552)	*P*_CAD_	*P* Ischemic stroke
Male/female	473/170	432/156	400/152	0.936	0.670
Age (years)	61.61 ± 11.95	62.32 ± 10.53	62.73 ± 12.37	0.270	0.111
Height (cm)	155.08 ± 7.84	164.11 ± 6.97	163.88 ± 7.12	0.000	0.000
Weight (kg)	54.56 ± 9.00	64.50 ± 10.68	63.14 ± 11.04	0.000	0.000
BMI (kg/m^2^)	22.66 ± 3.19	23.87 ± 3.24	23.44 ± 3.45	0.000	0.000
Smoking, *n* %	250 (38.8)	248 (42.2)	230 (41.7)	0.239	0.327
Alcohol, *n* %)	277 (43.1)	133 (22.6)	156 (28.3)	0.000	0.000
SBP (mmHg)	128.05 ± 19.00	132.93 ± 23.40	147.86 ± 21.89	0.000	0.000
DBP (mmHg)	80.41 ± 11.42	79.25 ± 14.15	83.84 ± 12.79	0.111	0.000
PP (mmHg)	47.64 ± 13.97	53.68 ± 17.54	64.02 ± 17.75	0.000	0.000
Glu (mmol/L)	6.01 ± 1.60	6.25 ± 1.48	6.20 ± 1.45	0.008	0.037
TC (mmol/L)	4.34 ± 1.05	4.55 ± 1.22	4.54 ± 1.16	0.001	0.002
TG (mmol/L)	1.38 ± 1.77	1.65 ± 1.11	1.68 ± 1.46	0.001	0.002
HDL-C (mmol/L)	1.90 ± 0.49	1.15 ± 0.34	1.23 ± 0.41	0.000	0.000
LDL-C (mmol/L)	2.72 ± 0.77	2.86 ± 1.02	2.83 ± 0.91	0.009	0.029
ApoA1 (g/L)	1.42 ± 0.27	1.03 ± 0.35	1.03 ± 0.23	0.000	0.000
ApoB (g/L)	0.90 ± 0.21	0.91 ± 0.27	0.89 ± 0.24	0.588	0.507
ApoA1/ApoB	1.65 ± 0.56	1.24 ± 0.80	1.25 ± 0.50	0.000	0.000

*SBP* Systolic blood pressure, *DBP* Diastolic blood pressure, *PP* Pulse pressure, *Glu* Glucose, *HDL-C* high-density lipoprotein cholesterol, *LDL-C* low-density lipoprotein cholesterol, *Apo* Apolipoprotein, *TC* Total cholesterol, *TG* Triglyceride. The value of triglyceride was presented as median (interquartile range), the difference between the control and CAD/Ischemic stroke groups was determined by the Wilcoxon-Mann-Whitney test

### Genotypic and allelic frequencies

The genotypic scattering of the *XKR6* rs7819412 SNP in both cases and controls conformed to Hardy-Weinberg equilibrium (*P* > 0.05). The genotypic as well as allelic frequencies of the rs7819412 SNP are represented in Table 2. The frequencies of the G and A alleles were 92.5 and 7.5% in controls; 89.9 and 10.1% in CAD patients (*P* = 0.024 vs. controls); and 90 and 10% in ischemic stroke patients (*P* = 0.036 vs. controls); respectively. The frequency of the GA/AA, GG genotypes were 14.3 and 85.7% in controls; 19 and 81% in CAD patients (*P* = 0.025 vs. controls); and 18.8 and 81.2% in ischemic stroke patients (*P* = 0.029 vs. controls); respectively.

**Table 2:** Genotype and allele frequencies of the *XKR6* rs7819412 SNP in cases and controls [*n* (%)]

Genotype/ Allele	Control (*n* = 643)	CAD (*n* = 588)	Ischemic stroke (*n* = 552)	CAD		Ischemic stroke	
				OR (95%CI)	*P*	OR (95%CI)	*P*
GG	551 (85.7)	476 (81.0)	447 (81.2)	1		1	
GA/AA	92 (14.3)	112 (19.0)	105 (18.8)	1.409 (1.042–1.906)	0.026	1.407 (1.036–1.911)	0.029
*x* ^2^	0.576	0.197	0.052				
HWE *(P)*	0.448	0.657	0.820				
*x* ^2^		4.991	4.794				
*P*		0.025	0.029				
G	1189 (92.5)	1057 (89.9)	994 (90.0)	1		1	
A	97 (7.5)	119 (10.1)	110 (10.0)	1.380 (1.042–1.827)	0.024	1.356 (1.019–1.805)	0.036
*x* ^2^		5.094	4.401				
*P*		0.024	0.036				

Adjusted for sex, age, smoking, drinking, BMI, diabetes, Mean arterial pressure, hyperlipidemia. *CAD* coronary artery disease

### *XKR6* rs7819412 SNP and the risk of CAD and ischemic stroke

The A allele was connected with high risk of CAD (adjusted OR = 1.38, 95% CI = 1.042–1.827) and ischemic stroke (adjusted OR = 1.365, 95% CI = 1.019–1.805; Table 2). The GA/AA genotypes were also related to an increased risk of CAD (adjusted OR = 1.409, 95% CI = 1.042–1.906) and ischemic stroke (adjusted OR = 1.407, 95% CI = 1.036–1.911). Unconditional logistic regression analysis showed that the subjects with GA/GG genotypes had high risk of CAD in the following subgroups: males (adjusted OR = 1.448, 95% CI = 1.034–2.141), BMI ≥ 24 kg/m^2^ (adjusted OR = 2.174, 95% CI = 1.227–3.756), and smokers (adjusted OR = 2.630, 95% CI = 1.593–4.342). The patients with GA/GG genotypes had high risk of ischemic stroke in the following subgroups: males (adjusted OR = 1.483, 95% CI = 1.023–2.148), BMI ≥ 24 kg/m^2^ (adjusted OR = 1.713, 95% CI = 1.048–2.858), and smokers (adjusted OR = 1.925, 95% CI = 1.137–3.257; Table 3). Some significant interactions were also detected in smoking, BMI ≥ 24 kg/m^2^ and genotypes.

**Table 3:** The risk of rs7819412 for CAD and ischemic stroke according to body mass index (BMI), gender, smoking and drinking

Factors	Genetype	CAD			Ischemic stroke		
		OR (95%CI)	*P*	*P interaction*	OR (95%CI)	*P*	*P interaction*
BMI							
< 24 Kg/m^2^	GG	1		0.000	1		0.006
	GA/AA	1.229 (0.841–1.795)	0.287		1.179 (0.793–1.753)	0.416	
≥ 24 Kg/m^2^	GG	1			1		
	GA/AA	2.174 (1.227–3.756)	0.007		1.731 (1.048–2.858)	0.032	
Gender							
Male	GG	1		0.058	1		0.735
	GA/AA	1.448 (1.034–2.141)	0.032		1.483 (1.023–2.148)	0.037	
Female	GG	1			1		
	GA/AA	1.250 (0.725–2.153)	0.422		1.243 (0.719–2.151)	0.436	
Smoking							
Nonsmoker	GG	1		0.000	1		0.014
	GA/AA	1.185 (0.813–1.727)	0.378		1.205 (0.823–1.765)	0.338	
Smoker	GG	1			1		
	GA/AA	2.630 (1.593–4.342)	0.000		1.925 (1.137–3.257)	0.015	
Drinking							
Nondrinker	GG	1		0.481	1		0.126
	GA/AA	1.238 (0.859–1.784)	0.253		1.266 (0.876–1.830)	0.210	
Drinker	GG	1			1		
	GA/AA	1.737 (0.993–3.037)	0.053		1.514 (0.943–2.672)	0.152	

*CAD* coronary artery disease

### Related risk factors for CAD and ischemic stroke

Unconditional logistic regression analysis revealed that the incidence of CAD and ischemic stroke was positively correlated with hyperlipidemia, BMI, hypertension, smoking, diabetes, and the rs7819412 GA/AA genotypes and negatively correlated with alcohol consumption (Table 4).

**Table 4:** The relative risk factors for CAD and Ischemic stroke

Factor	CAD		Ischemic stroke	
	OR (95%CI)	*P*	OR (95%CI)	*P*
BMI < 24 kg/m^2^	1		1	
BMI ≥ 24 kg/m^2^	1.417 (1.118–1.795)	0.004	1.542 (1.204–1.975)	0.001
Nonsmoking	1		1	
Smoking	1.306 (1.020–1.674)	0.034	1.477 (1.136–1.920)	0.004
Nondrinking	1		1	
Drinking	0.556 (0.424–0.728)	0.010	0.414 (0.316–0.544)	0.000
Rs7819412GG	1		1	
Rs7819412GA/AA	1.533 (1.131–2.077)	0.006	1.461 (1.067–2.001)	0.018
Non-diabetes	1		1	
Diabetes	1.476 (1.080–2.018)	0.015	1.486 (1.117–1.977)	0.006
Normotensive	1		1	
Hypertension	1.192 (0.889–1.600)	0.240	1.250 (0.974–1.604)	0.079
Normal serum lipids	1		1	
Hyperlipidemia	1.439 (1.133–1.828)	0.003	1.582 (1.219–2.053)	0.001

### Genotypes and serum lipid levels

The *XKR6* rs7819412A allele carriers had higher serum TC levels in ischemic stroke and higher serum TG levels in CAD patients than the rs7819412A allele non-carriers (*P* < 0.05; Table 5).

**Table 5:** Association of the genotypes and serum lipid levels in controls and CAD and ischemic stroke patients

Genotype	*n*	TC (mmol/L)	TG (mmol/L)	HDL-C (mmol/L)	LDL-C (mmol/L)	ApoA1 (g/L)	ApoB (g/L)	ApoA1/ ApoB
Control								
GG	551	4.35 ± 1.04	1.35 ± 1.70	1.90 ± 0.49	2.74 ± 0.76	1.42 ± 0.28	0.91 ± 0.21	1.66 ± 0.58
GA/AA	92	4.25 ± 1.07	1.54 ± 2.11	1.85 ± 0.50	2.61 ± 0.87	1.38 ± 0.26	0.88 ± 0.21	1.63 ± 0.40
*F*		1.306	0.987	0.941	2.276	0.924	0.444	0.228
*P*		0.254	0.321	0.332	0.132	0.337	0.505	0.633
CAD								
GG	476	4.55 ± 1.19	1.59 ± 1.00	1.15 ± 0.34	2.82 ± 0.99	1.02 ± 0.32	0.91 ± 0.26	1.23 ± 0.84
GA/AA	112	4.57 ± 1.33	1.92 ± 1.48	1.15 ± 0.34	3.00 ± 1.16	1.06 ± 0.45	0.90 ± 0.28	1.27 ± 0.58
*F*		0.004	7.267	0.100	2.719	1.411	0.491	0.344
*P*		0.952	0.007	0.752	0.100	0.235	0.484	0.558
Ischemic stroke								
GG	447	4.49 ± 1.15	1.64 ± 1.24	1.23 ± 0.42	2.81 ± 0.87	1.02 ± 0.22	0.89 ± 0.24	1.25 ± 0.47
GA/AA	105	4.73 ± 1.21	1.85 ± 2.17	1.27 ± 0.37	2.92 ± 1.07	1.05 ± 0.26	0.93 ± 0.27	1.26 ± 0.60
*F*		4.270	1.672	0.887	1.822	0.985	3.254	0.000
*P*		0.039	0.197	0.347	0.178	0.321	0.072	0.993

Adjusted for sex, age, smoking, drinking, BMI, diabetes, hypertension, hyperlipidemia. *TC* total cholesterol, *TG* triglyceride, *HDL-C* high-density lipoprotein cholesterol, *LDL-C* low-density lipoprotein cholesterol, *ApoA1* apolipoprotein A1, *ApoB* apolipoprotein B, *CAD* Coronary artery disease

### Relative factors for serum lipid parameters

**Fig. 1:**
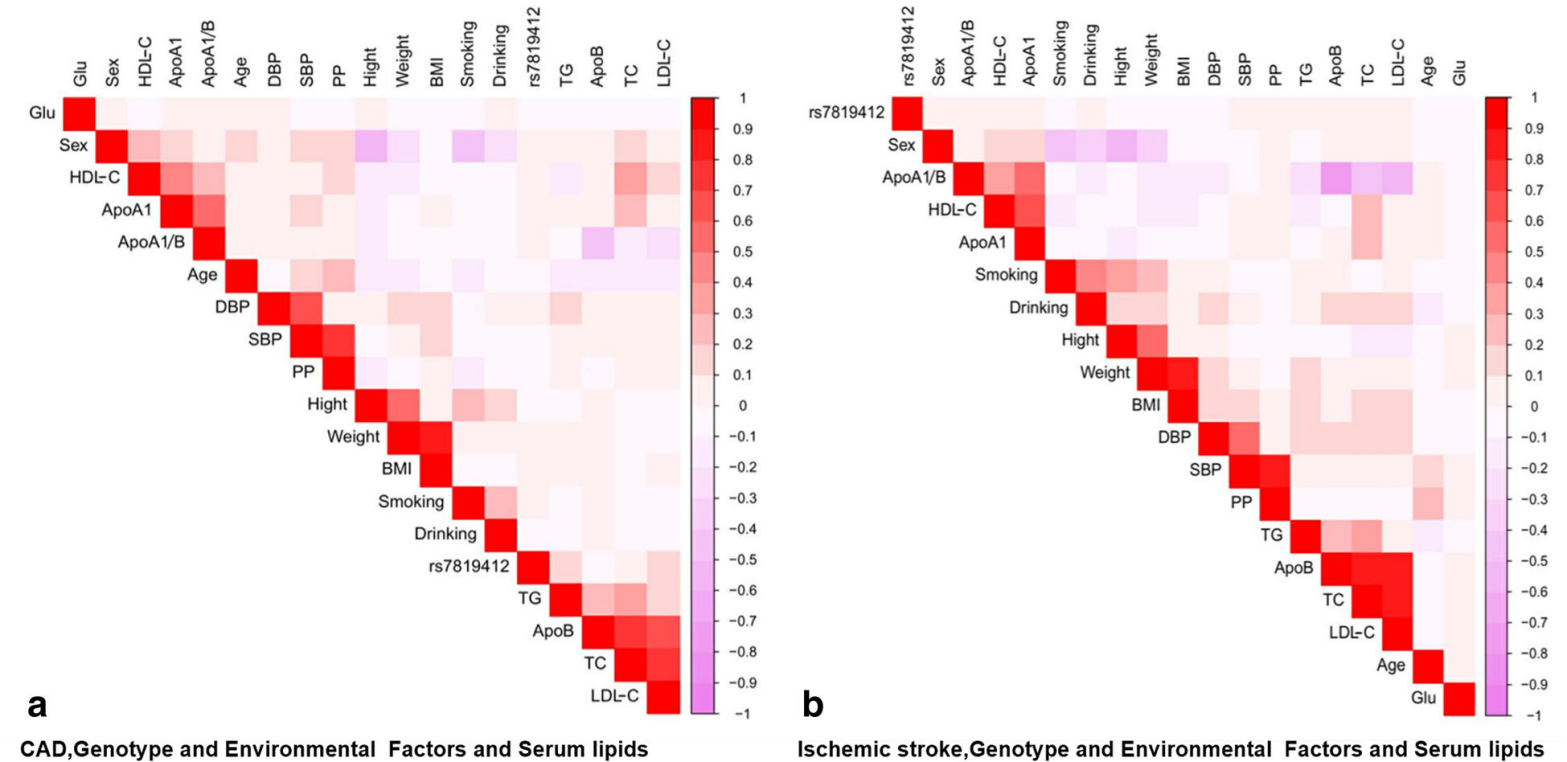
Correlation among environmental exposures, the *XKR6* rs7819412 SNP and serum lipid variables in the patients with CAD (**a**) or ischemic stroke (**b**). *CAD* Coronary artery disease; *TC* total cholesterol; *TG* triglyceride; *HDL-C* high-density lipoprotein cholesterol; *LDL-C* low-density lipoprotein cholesterol; *ApoA1* apolipoprotein A1; *ApoB* apolipoprotein B; *ApoA1/B* the ratio of apolipoprotein A1 to apolipoprotein B; *BMI* body mass index

As shown in Fig. 1, Pearson correlation analysis suggested that the rs7819412 SNP was connected with serum lipid levels, and several environmental factors such as sex, age, alcohol consumption, cigarette smoking, BMI and blood pressure levels were also correlated with serum lipid parameters in both patient groups.

## Discussion

A previous study suggested that the incidence and progress of CAD were influenced by both environmental and genetic factors and their interactions [26]. Hundreds of GWASes indicated that lots of SNPs have been related to some risk factors that could contribute to the development of CAD, such as obesity, serum lipid levels and hypertension [27–29]. Recent research suggested that a healthy lifestyle among individuals with high genetic risk profile could reduce the relative risk of CAD by nearly 50% compared with a poor lifestyle, suggesting that genotyping and early lifestyle intervention could effectively reduce the incidence of CAD in high-risk group [30]. The current study showed that the allelic and genotypic frequencies of the rs7819412 SNP were markedly different between controls and patients with ischemic stroke or CAD, and the A allele and GA/AA genotypes were associated with an increased risk of ischemic stroke and CAD in the Han Chinese. In other words, the rs7819412 SNP may be a genetic risk factor for ischemic stroke and CAD.

Previous work demonstrated that the rs7819412 SNP was linked to increased TG levels and T2DM in Europeans [16, 31]. A lot of studies showed that hyperlipidemia and T2DM were the severe risk factors for CAD and ischemic stroke, these risk factors are evenly related to the increased incidence of both diseases [32–35]. Thus, we speculated that the rs7819412 SNP might be related to the incidence of ischemic stroke and CAD. In the current research, we noticed that the patients with A allele had increased TG levels in CAD and TC levels in ischemic stroke patients, and we also found that the GA/AA genotypes and A allele were connected with high risk of CAD and ischemic stroke.

In the present study, we also found that the subjects with GA/AA genotypes had an increased risk of CAD in the following subgroups: males, high BMI and smokers. It is well-known that people with high BMI had higher mortality and incidence of cardiac events [36]. Several previous studies have proved that obesity is a shared and most important risk factor for several different types of cardiovascular and cerebrovascular diseases, such as heart failure, ischemic stroke and CAD [37–39]. Furthermore, it has been noticed that there are significant differences in height, weight, and mass and distribution of fat between males and females. It is essential to take these differences into consideration in assessing the meaning of cardiac symptoms between men and women, and these differences indicate that there may be a sex-specific in the occurrence and development of CAD [40]. Gender is considered as a self-determining risk element for dyslipidemia, cardiovascular disease and ischemic stroke. In addition, the influence of smoking on dyslipidemia has attracted more and more attention. Several recent studies noted that the lower HDL-C levels and increased ratio of the ApoA1/ApoB and serum TG, LDL-C, TC levels in smokers compared to non-smokers, all of these were related to the development of CAD and ischemic stroke [41–43]. In our research, the interaction between the *XKR6* rs7819412 SNP and gender, high BMI and smoking was found and the risk of CAD and ischemic stroke was also increased.

It is now generally accepted that the cholesterol-lowering action of lipid-lowering drugs is the most important factor to reduce the occurrence of adverse events and mortality in patients with CAD and ischemic stroke [44]. But, genotyping of high-risk population could produce great significance for the early prevention of CAD and ischemic stroke, and it has also attracted more attentions. In our research, we proved that the *XKR6* rs7819412 SNP not only contributed to serum lipid levels and increased the risk of CAD and ischemic stroke, but also interacted with several environment factors. Therefore, the *XKR6* rs7819412 SNP may be a new target for early prevention and treatment of hyperlipidemia and atherosclerosis-related diseases.

## Limitations

This study may have several limitations. Firstly, the number of cases was relatively small as compared to other studies. Thus, a study with larger sample size was needed to demonstrate our results. Secondly, many patients were taking some drugs that were used for secondary prevention of CAD. All drugs have some certain impacts on serum lipid levels. Thirdly, although several environmental factors such as sex, age, blood pressure, cigarette smoking, BMI and alcohol consumption have been adjusted for the statistical analysis, some general characteristics were different between controls and cases. Finally, in spite of we noticed that the rs7819412 A allele was connected with increased serum TG, TC levels and the risk of CAD and ischemic stroke, in order to further clarify the mechanism, some efficient studies should be carried out.

## Conclusions

The results of the present study showed that the genotypic and allelic frequencies of the *XKR6* rs7819412 SNP were obviously different between controls and patients with CAD and ischemic stroke. The GA/AA genotypes were associated with increased serum TG levels in CAD and TC levels in ischemic stroke patients. The patients with GA/AA genotypes or A allele had an increased risk of CAD and ischemic stroke in the following subgroups: males, BMI ≥ 24 kg/m^2^ and smokers.

## Data Availability

The datasets generated during the present study are not publicly available, because detailed genetic information of each participant was included in these materials.

## Abbreviations

ANCOVA: Covariance analysis
Apo: Apolipoprotein
BMI: Body mass index
CAD: Coronary artery disease
CI: Confidence interval
DNA: Deoxyribonucleic acid
GWAS: Genome-wide association study
HDL-C: High-density lipoprotein cholesterol
LDL-C: Low-density lipoprotein cholesterol
OR: Odds ratio
SNP: Single nucleotide polymorphisms
T2DM: Type 2 diabetes mellitus
TC: Total cholesterol
TG: Triglyceride
XKR6: XK related 6
